# SXT/R391 integrative and conjugative elements in *Proteus* species reveal abundant genetic diversity and multidrug resistance

**DOI:** 10.1038/srep37372

**Published:** 2016-11-28

**Authors:** Xinyue Li, Yu Du, Pengcheng Du, Hang Dai, Yujie Fang, Zhenpeng Li, Na Lv, Baoli Zhu, Biao Kan, Duochun Wang

**Affiliations:** 1 National Institute for Communicable Disease Control and Prevention, China CDC/State Key Laboratory of Infectious Disease Prevention and Control, Beijing, China; 2 Collaborative Innovation Center for Diagnosis and Treatment of Infectious Diseases, Hangzhou, China; 3 Institute of Infectious Diseases, Beijing Ditan Hospital, Capital Medical University, Beijing, China; 4 Institute of Microbiology, Chinese Academy of Sciences, Beijing, 100101 PR China

## Abstract

SXT/R391 integrative and conjugative elements (ICEs) are self-transmissible mobile genetic elements that are found in most members of *Enterobacteriaceae*. Here, we determined fifteen SXT/R391 ICEs carried by *Proteus* isolates from food (4.2%) and diarrhoea patients (17.3%). BLASTn searches against GenBank showed that the fifteen SXT/R391 ICEs were closely related to that from different *Enterobacteriaceae* species, including *Proteus mirabilis*. Using core gene phylogenetic analysis, the fifteen SXT/R391 ICEs were grouped into six distinct clusters, including a dominant cluster and three clusters that have not been previously reported in *Proteus* isolates. The SXT/R391 ICEs shared a common structure with a set of conserved genes, five hotspots and two variable regions, which contained more foreign genes, including drug-resistance genes. Notably, a class A β-lactamase gene was identified in nine SXT/R391 ICEs. Collectively, the ICE-carrying isolates carried resistance genes for 20 tested drugs. Six isolates were resistant to chloramphenicol, kanamycin, streptomycin, trimethoprim-sulfamethoxazole, sulfisoxazole and tetracycline, which are drug resistances commonly encoded by ICEs. Our results demonstrate abundant genetic diversity and multidrug resistance of the SXT/R391 ICEs carried by *Proteus* isolates, which may have significance for public health. It is therefore necessary to continuously monitor the antimicrobial resistance and related mobile elements among *Proteus* isolates.

Integrative and conjugative elements (ICEs) are self-transmissible mobile genetic elements that can be excised from the chromosome of the host cell. Once excised, ICEs form a circular intermediate that can be transferred to another cell via conjugation^1^. Many varieties of ICEs have been found in diverse Gram-positive and Gram-negative bacteria^2^,^3^,^4^,^5^.

The SXT/R391 ICE family is one of the largest ICE families, with the most abundant diversity and members among Gram-negative bacteria^6^. SXT was first discovered in MO10, which is a *Vibrio cholerae* O139 clinical strain isolated from India in the early 1990s. SXT^MO10^ is an ~100 kb ICE that carries genes encoding resistance to sulfamethoxazole, trimethoprim, chloramphenicol, and streptomycin^7^. Since then, ICEs related to SXT^MO10^ have been detected in most *Vibrio* species in addition to *V. cholerae* as well as in other gammaproteobacteria^8^,^9^. R391 was first discovered in a *Providencia rettgeri* clinical isolate from South Africa in 1967^10^; subsequent studies showed that it genetically and functionally belonged to the SXT family^11^. The R391 ICE mediates resistance to kanamycin and the heavy metal Hg^11^.

Comparative genomics has shown that the SXT/R391 ICEs share nearly identical sets of 52 conserved core genes that are involved in integration/excision, conjugative transfer, and regulation^12^. All SXT/R391 ICEs detected to date have element-specific phenotypes that are conferred by the insertion of variable DNA sequences into several sites. Variable DNA sequences are frequently found in five hotspots (HS1–HS5) and four variable regions (VRI-IV)^12^. The hotspots are sites within the conserved SXT/R391 ICE backbone have variable DNA present in all of the ICEs, which inserted into their intergenic regions that confer element-specific properties, these variable DNA sequences share a mosaic structure and have sizes ranging from 30–60 kb. Except for the hotspots, some SXT/R391 ICEs also contain variable regions, which encode resistance to antibiotics, heavy metals and quaternary ammonium compounds^12^,^13^.

The genus *Proteus* is a motile Gram-negative bacterium that survives in soil, water, and the intestinal tracts of mammals; bacteria of this genus belong to the family *Enterobacteriaceae. Proteus* consists of five species and three unnamed genomospecies^14^. Among these species, *P. vulgaris* and *P. mirabilis* are most frequently linked with food contamination and food poisoning. These species are also renowned opportunistic pathogens that cause a variety of infections in humans, including respiratory tract, wound, burn, skin, eye, ear, nose, and throat infections^15^.

In previous studies, a novel SXT/R391-related ICE (ICE*Pmi*Jpn1) carrying *bla*_*CMY*-*2*_ on the chromosome of a *P. mirabilis* clinical isolate was found and characterized. ICE*Pmi*Jpn1 was the first ICE identified to confer resistance to extended-spectrum cephalosporins^16^. Recently, Lei, *et al.* reported SXT/R391 ICEs in *P. mirabilis* isolates from food-producing animals in China^17^. However, our knowledge of the prevalence and origin of the *Proteus* species ICEs is limited. In this study, we screened SXT/R391-specific genes from 123 *Proteus* isolates collected from clinical and food samples from 2008 to 2015 in China. We detected 15 *Proteus* isolates that were positive for SXT/R391-related ICEs and analysed the genetic structure and evolutionary origins of each SXT/R391 ICE. Additionally, we determined their transfer capabilities and the roles of the SXT/R391 ICEs in drug resistance.

## Materials and Methods

### Ethics

The study was approved by the Ethics Committee of National Institute for Communicable Disease Control and Prevention, Chinese Center for Disease Control and Prevention, and the study was carried out in accordance with the approved guidelines. Informed consents were obtained from all the patients.

### Collection of samples, bacterial isolation and identification of *Proteus* isolates

A total of 123 *Proteus* isolates were included in this study. The strains were isolated from stool samples and/or rectal swabs from diarrhoea patients (n = 75) and fresh food samples (n = 48) collected from 2008 to 2015 in three cities (Beijing, Tianjin and Ma’anshan) in China. Specimens of diarrhoea patients were collected in Cary-Blair transport media, and then each sample was incubated for 24 h at 37 °C on Salmonella-Shigella (SS) and MacConkey agar (Becton Dickinson Co., USA). Suspicious colonies were streaked on nutrient agar for incubation (37 °C, 24 h), single clone from nutrient agar was then picked for biochemical identification. The fresh food samples included meat of raw pork, beef, chicken, duck, fish and shrimp, 25 gram each food sample was put into 225 ml of Gram negative enrichment broth (Qingdao Hopebio Technology Co., Ltd, China) and enriched for 8 h at 37 °C, then streaked on SS and MacConkey agar, followed by the procedure likes that sample of diarrhoea patient. All isolates from nutrient agar were preliminarily identified as *Proteus* by negative for oxidase and positive for urease and phenylalanine deaminase and KIA: K/A++. The isolates were confirmed to be *P. mirabilis* (n = 81) and *P. vulgaris* (n = 42) by API20E biochemical test (BioMerieux, Lyon, France). All isolates were stored at −70 °C in LB broth containing 15% glycerol prior to use.

### PCR screening of SXT/R391 ICEs and genomic sequencing

All 123 *Proteus* strains in this study were screened for the presence of SXT/R391-like ICEs using a PCR-based method targeting the *int*_SXT_ gene^18^, which encodes a conserved SXT/R391 ICE integrase. Next-generation sequencing (NGS) was performed with the PCR-positive isolates. Genomic DNA was extracted from 5 ml of overnight cultures using a Wizard Genomic DNA Purification kit (Promega, USA) according to the manufacturer’s instructions. The extracted DNA was dissolved in Tris-EDTA buffer and stored at −20 °C prior to sequencing. The genomes were commercially sequenced using Illumina HiSeq 2000 sequencing (Illumina Inc., San Diego, CA, USA) by constructing two paired-end libraries with average insert lengths of 500 bp and 2000 bp. Then, 100× libraries were obtained with clean paired-end read data. Assembly was performed using SOAP denovo v2.04^19^.

### Extraction, assembly and annotation of the SXT/R391 ICEs

The contigs of the fifteen SXT/R391 ICEs were extracted and assembled from whole sequenced genomes using SOAP denovo v2.0.4^19^ against the reference SXT/R391 ICEs in the genome of *P. mirabilis* HI4320 (accession number: AM942759.1). The relationships between contigs were displayed using ContigScape^20^ with custom primer walking. Sanger sequencing was used to close the gaps in the ICE region and the results were verified by PCR. The Phred/Phrap/Consed software was used for primer design, genome assembly, editing and quality assessment (http://www.phrap.org/consed/consed.html). Regions with low quality of the genome were resequenced. Putative functions were inferred using the Basic Local Alignment Search Tool (BLAST) (http://ncbi.nlm.nih.gov/BLAST) and the ORF finder (http://www.ncbi.nlm.nih.gov/projects/gorf). The RAST (Rapid Annotation using Subsystem Technology, version 4.0)^21^,^22^,^23^ server pipeline was used to predict open reading frames (ORFs) and annotate the ORFs of the recovered ICEs.

### Phylogenetic and structural analysis of the SXT/R391 ICEs

To investigate the evolutionary origins of the SXT/R391 ICEs in *Proteus* species, we performed a phylogenetic analysis. First, we used each of the fifteen SXT/R391 ICEs in a BLASTn search to obtain all high homology ICEs in the public database (last updated in Nov 2015). Then, we scanned and selected 24 ICEs as references, representative for the different evolutionary origins and species based on different scores and identifications (Supplementary Table S1). To construct the phylogenetic tree, core genes of all ICEs were identified using the OrthoMCL software. The concatenated sequences of these core genes were used for phylogenetic analysis. The bootstrap values were calculated based on 1000 replicates. The structures of the fifteen SXT/R391 ICEs were constructed by comparison with the reference ICE in the genome of *P. mirabilis* HI4320 (AM942759.1) using BLAST and SOAPdenovo 2.04^19^. The tested ICEs were compared against the reference ICE from *P. mirabilis* HI4320 using Pheatmap (R package version 0.7.7) to plot heatmaps of the annotated core and pan genes of each ICE.

### Antimicrobial susceptibility testing

Antimicrobial susceptibility testing was performed on *P. mirabilis* by broth dilution method according to the guidelines for MIC testing from the Clinical and Laboratory Standards Institute (CLSI) (2015). Twenty antimicrobials were tested as follows: ampicillin (AMP), cefazolin (CFZ), cefuroxime (CXM), ceftriaxone (CRO), cefepime (FEP), cefotetan (CTT), aztreonam (ATM), imipenem (IPM), ampicillin-sulbactam (SAM), piperacillin-tazobactam (TZP), amikacin (AMK), gentamycin (GEN), kanamycin (KAN), streptomycin (STR), sulfamethoxazole (SUL), trimethoprim-sulfamethoxazole (SXT), ciprofloxacin (CIP), chloramphenicol (CHL), tetracycline (TCY), and azithromycin (AZM). The CLSI(2015) breakpoints were used to determine susceptibility and resistance.

### Conjugation experiments

A mating assay was employed to test ICE mobility. The experiment was conducted as previously described^24^. Transconjugants were selected on LB agar plates containing streptomycin (100 μg/ml) and kanamycin (100 μg/ml). The ICE transfer frequency was expressed as the number of transconjugants observed per recipient cell (*E. coli* SM10). Transconjugants were confirmed by PCR detection of *int*_SXT_ and antibiotic resistance genes specific for each of the SXT/R391 ICEs. The primer information is listed in Supplementary Table S2.

### Sequence data access

The reads sequences and annotated genes of the fourteen ICEs (except for ICE*Pmi*CHN3277) in *Proteus* species were submitted to GenBank under accession number KX243403- KX243416.

## Results

### Distribution and general features of SXT/R391 ICEs among *Proteus* strains

Fifteen out of 123 strains (12.2%, fourteen *P. mirabilis* and one *P. vulgaris* strain) were positive for the *int*_SXT_ gene, including 4.2% (2/48) of food samples (crab and pork), and 17.3% (13/75) of stool samples from the diarrhoea patients, respectively. The genomes of the 15 *int*_SXT_-positive strains were obtained and all SXT/R391 ICEs were successfully assembled with the exception of the ICE from isolate TJ3277. The general genomic features of the fifteen ICEs are summarized in Table 1. The SXT/R391 ICEs were designated according to the nomenclature proposed for this family of elements^2^. The lengths of the 15 ICEs ranged from 76,218 bp (ICE*Pmi*CHN1809) to 108,335 bp (ICE*Pmi*CHN3300), with an average length of 93,396 bp. The G + C content ranged from 44.6% (ICE*Pmi*CHN1586) to 47.8% (ICE*Pmi*CHN905/ICE*Pmi*CHN3335) among the SXT/R391 ICEs, with a mean of 46.9%. The total numbers of predicted CDSs were between 53 and 99.

### Blast searches of closely related SXT/R391 ICEs in GenBank

As shown in Table 2, the 15 SXT/R391 ICEs were closely related to many different ICEs based on some indicators, such as the highest max score, query coverage and identity. These ICEs derived from *Alteromonas macleodii*, *Alteromonas mediterranea*, *Vibrio cholerae*, *Vibrio alginolyticus*, *Proteus mirabilis* and *Providencia stuartii*. Due to the closely related scores among the hits of each ICE, we listed the top three alignment results for each ICE; the max score ranged from 19,287 to 1.36E + 05, the total score (bits) ranged from 73,099 to 1.95E + 05, the highest query ranged from 46% to 99% and the identity ranged from 96% to100%. Notably, ICE*Pmi*CHN3335 (host strain TJ3335, isolated from the stool of a patient from Tianjin city, see Table 1) was closely related to ICE*Pmi*Chn1 (host strain PM13C04, isolated from a chicken in Hubei, China, in 2013, see Supplementary Table S1) with a highest query and identity of 99%. However, some ICEs, such as ICE*Pmi*CHN2407, ICE*Pmi*CHN2410 and ICE*Pmi*CHN2416, only exhibited query coverage from 46% to 67% and identity from 96% to 99%.

### Phylogenetic analysis of ICEs in *Proteus* isolates

**Figure 1:**
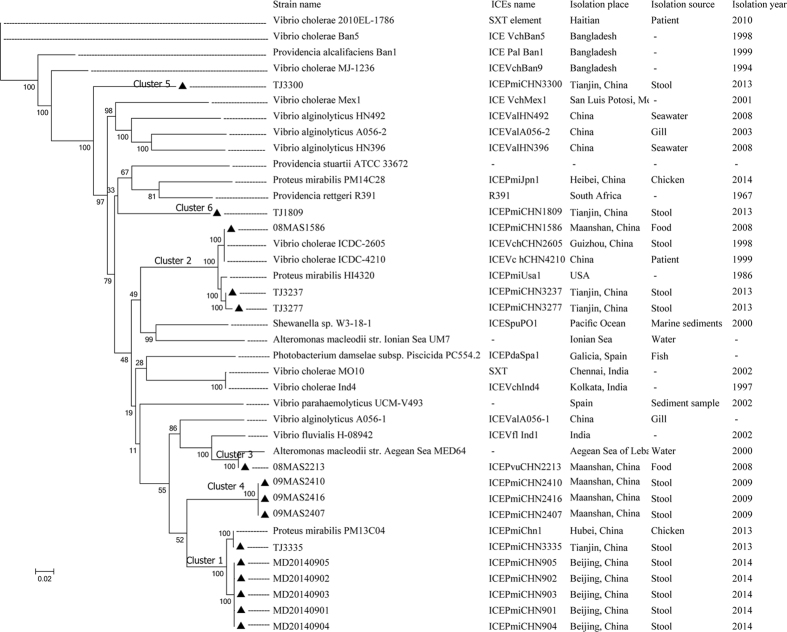
Phylogenetic tree from the maximum-likelihood analysis of the core genome alignments of the fifteen ICEs (black triangle). The scale bar indicates substitutions per site. Bootstraps are indicated at each node.

The fifteen SXT/R391 ICEs were grouped into six clusters based on the core gene phylogenetic analysis (Fig. 1). Six ICEs, their contained strains isolated from the stool of diarrhoeal patients in two cities (Table 1), belonged to cluster I, with the reference ICE*Pmi*Chn1, which was an ICE contained in a *P. mirabilis* strain isolated from a chicken in Hubei, China (Supplementary Table S1). Three ICEs (strains isolated from food and stool samples from diarrhoea patients in two cities in China) belonged to cluster II, with references including ICEs from *V. cholerae* strain ICDC-4210 (isolated from the stool of a patient in Jiangxi, China) and *P. mirabilis* strain HI4320. Cluster III contained ICE*Pvu*CHN2213 from a *P. vulgaris* strain isolated from food that was closely related to the reference ICE of *Alteromonas macleodii* MED64 (from waters off the Aegean Sea)^25^. The remaining five ICEs were grouped into three distinct clusters (IV, V and VI) with no references; thus, these ICEs were designated as novel ICEs.

### Sequence structure and variable region characteristics of the SXT/R391 ICEs

**Figure 2:**
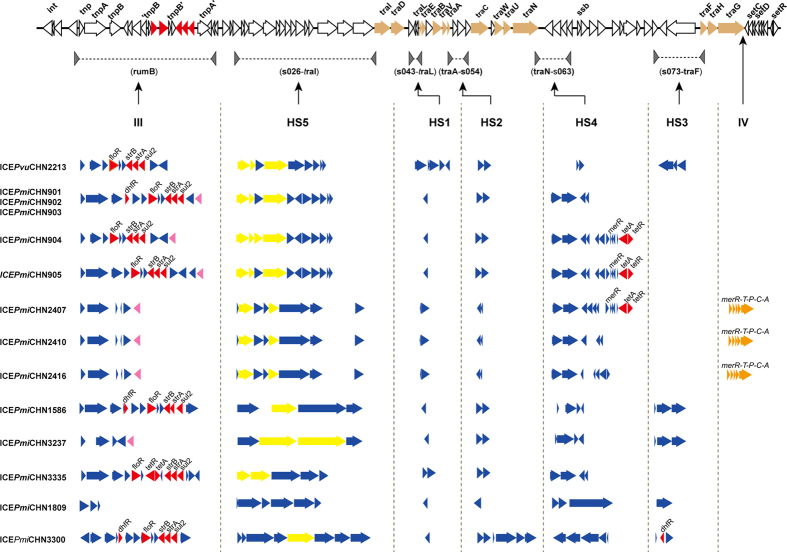
Genetic organization of the ICEs in this study. The upper indicate backbone, represents the 52 conserved core genes of the SXT/R391 family ICEs. Below the common structure, indicate five hotspots (HS1–5) and two variable (III and IV) regions of the fourteen ICEs (except for ICE*Pmi*CHN3277, which was incompletely assembled). Drug-resistance gene (red triangle); the restriction-modification system (RM) (yellow triangle); β-lactamase encoding gene (pink triangle); mercury resistance gene (orange triangle); other genes (blue triangle). Detailed annotated genes from all fifteen ICEs are listed in Supplementary Table S3.

Except for SXT/R391 ICE from TJ3277, which was partially assembled, the remaining fourteen SXT/R391 ICEs shared a common structure that was identical to most SXT/R391 ICEs. In addition to the common structure, all fourteen ICEs contained five hotspots (HS1–5) and two variable (III and IV) regions (Fig. 2). VRIII, inserted into *rumB* gene, was found in all 14 SXT/R391 ICEs. Five ICEs (ICE*Pmi*CHN901, ICE*Pmi*CHN902, ICE*Pmi*CHN903 ICE*Pmi*CHN1586 and ICE*Pmi*CHN3300), contained the *dhfR*, *floR*, *strB*/*A* and *sul2* genes in this region, which conferred resistance to SXT, CHL, STR and SUL, respectively. The *tetA* and *tetR* tetracycline resistance genes were present in VRIII of ICE*Pmi*CHN3335 and HS4 of ICE*Pmi*CHN904 and ICE*Pmi*CHN904. Notably, nine ICEs contained a β-lactamase gene in VRIII (Fig. 2 and Supplementary Table S3), which showed 100% identity to the class A β-lactamase gene bla (HMS-1) contained in plasmid R997 from *P. mirabilis* (GenBank: KX228735) and *V. parahaemolyticus* strain UCM-V493 (CP007004.1). HS1, HS2, HS4 and HS5 were detected in all ICEs. However, HS3 was detected in only five ICEs with few inserted genes; a *dhfR* gene was located in HS3 of ICE*Pmi*CHN3300. Abundant foreign genes were inserted into HS4 and HS5, including genes encoding the restriction-modification system (RM) of which the Type I RM was common, serine protease, ATPase, helicase, and exonuclease. Additionally, genes encoding hypothetical proteins with unknown functions were frequently inserted into these two regions. In VRIV, a *mer* operon was found in ICE*Pmi*CHN2407, ICE*Pmi*CHN2410 and ICE*Pmi*CHN2416, this *mer* operon was also reported at ICEs in bacterial strains from aquaculture environments, previously described for R391 ICEs and mediated resistance to mercury^13^. The annotated genes from all fifteen ICEs and the six closely related ICEs (Supplementary Table S1) were used to construct a heatmap using *P. mirabilis* HI4320 (AM942759.1) as the reference; ICE*Pmi*CHN3277 was excluded because it was incompletely assembled. The remaining fourteen ICEs shared minor numbers of common genes (Supplementary Fig. S1) between each of their ICEs and the closely related ICEs (e.g., ICE*Pmi*CHN1809 vs the ICE of *Providencia stuartii* strain ATCC33672, ICE*Pmi*CHN1586 vs ICE*Vch*CHN2605, and ICE*Pmi*CHN3237 and ICE*Pmi*CHN3277 vs ICE*Vch*CHN4210 had extensive diversity in their annotated genes).

### Antibiotic resistance of the *Proteus* isolates and their relationship with ICEs

Phenotypically, all fifteen SXT/R392 ICE-harbouring isolates presented multi-drug resistance to the 20 tested drugs (Table 1). Isolates MD20140901, MD20140904 and MD20140905 with the most antibiotic phenotypes were resistant to 16 out of 20 drugs. Even the least resistant isolate (TJ3300) was resistant to five drugs. 09MAS2407 and 09MAS2410, MD20140901 and MD20140905, and TJ3237 and TJ3277 shared same multidrug resistance patterns, respectively. Notably, all isolates were resistant to AMP and 12 isolates (in addition to TJ1809, TJ3237 and TJ3277) were resistant to CHL, STR, SXT and SUL, which are commonly encoded by SXT/R391 ICEs. MD20140901 to MD20140905 were resistant to the first, second and third-generation cephalosporins; MD20140904 was also resistant to the fourth generation cephalosporin.

All isolates positive for resistant related genes (*floR*, STR, SUL and SXT) at ICEs, were phenotypic resistant to their drugs (chloramphenicol, streptomycin, sulfisoxazole and trimethoprim-sulfamethoxazole), by contrast, even if isoates were phenotypic resistant to those four drugs, their ICEs not always carried those resistant related genes (Table 1 and Fig. 2).

### Transfer ability

To test the transfer ability of the *Proteus* isolate ICEs, we selected five ICE-carrying isolates (08MAS2213, 08MAS1586, 09MAS2407, TJ1809, and TJ3335) for the mobility test. Transconjugants were obtained with a transfer frequency of 5.0 × 10^−6^ (TJ3335) to 2.5 × 10^−2^ (08MAS1586) per recipient cell (Table 1). The transconjugants were confirmed by PCR detection of the *int*_*SXT*_ and antibiotic resistance genes (*strA*/*B*, *sul2*, *floR*, and *dfrA*) in the recipient cells.

## Discussion

In this study, the ICEs contained in *Proteus* isolates showed high diversity compared to those carried in a variety of other bacterial species. Our results indicated that SXT/R391 ICEs presented a strong ability to transmit among different bacterial species as a type of self-transmissible mobile genetic element. This study revealed the epidemiology of the spatio-temporal prevalence of ICEs in *Proteus*. The occurrence and dispersion of *Proteus* isolates from different regions in China revealed the occurrence and widespread distribution of ICEs among *Proteus*. Furthermore, the various ICEs conferred phenotypes such as multidrug and heavy metal resistance to their host strains. To date, at least 89 SXT/R391-family ICEs have been identified (http://db-mml.sjtu.edu.cn/ICEberg/)^26^. Most ICEs have been investigated in *V. cholerae*^4^,^27^,^28^,^29^,^30^,^31^, which is the aetiological agent of the diarrhoeal disease cholera. The ICEs reported in *Proteus* strains including R997 (India)^32^, ICE*Pmi*USA1 (America)^33^, ICE*Pmi*Jpn1(Japan)^16^, ICE*Pmi*Spn1(Spain)^24^ and ICE*Pmi*Chn1(China)^17^. In this study, our fifteen ICEs revealed six distinct clusters and were positioned in different branches of the phylogenetic tree. The Blastn analysis in Genbank showed that closely related ICEs might be different. Additionally, five of our ICEs grouped into three distinctive clusters representing novel ICEs (Fig. 1), which increases the number of the SXT/R391 family members. Consequently, our results indicated that abundant genetic diversities and variable types of ICEs were ubiquitous in *Proteus* strains, compared with the ICEs in *V. cholerae* and other *Enterobacteriaceae*. The ICE types in the *Proteus* strains in our study were different from a recent investigation of the SXT/R391 ICEs in *P. mirabilis* isolates from food-producing animals in China^17^, which included two types of ICEs (ICE*Pmi*Jpn1 and ICE*Pmi*Chn1). We suggest that the use of a more extensive source of isolates (food and clinical samples from different cities) might contribute to the varieties of ICEs in *Proteus* isolates. Regardless of the diversity of the hosts and locations, the ICEs of the SXT/R391 family share a common structure and contain 52 conserved core genes that mediate integration, recombination, DNA repair and conjugative transfer^12^. In this study, the ICE-harbouring *Proteus* strains shared a common structure even though they were isolated from different cities in China and were at different evolutionary stages. However, the 15 ICEs were closely related to many different ICEs derived from *Alteromonas macleodii*, *A. mediterranea*, *V. cholerae*, *V. alginolyticus*, *P. mirabilis* and *Providencia stuartii*. They share much different query score and identity at nucleotide level (Table 1), consequently, these ICEs formed unique variable regions. Antibiotic resistance genes were typically present within VRIII in ICEs reported in previous studies. Our results showed that most of the multidrug resistance genes were present in this region, including the genes encoding resistance for sulfamethoxazole, trimethoprim, chloramphenicol, and streptomycin that were first described in the SXT of *V. cholerae* O139 MO10^7^. A class A β-lactamase gene was found, which has been reported in the plasmid from *P. mirabilis* (GenBank: KX228735), and the ICE from *V. parahaemolyticus*^34^. However, further research will be necessary to confirm the relationship of this gene with resistance to β-lactams. Five hotspots were located in *s043*-*traL* (HS1), *traA*-*s054* (HS2), *s073*-*traF* (HS3), *traN*-*s063* (HS4), and *s026*-*traI* (HS5). Variable genes were identified and predicted to encode restriction-modification systems, endonucleases, which may provide protection from invasion by foreign DNA^12^. ICEs without any antibiotic resistances were described^12^ and determinants for antibiotic resistance were not found in ICE*Pmi*CHN1809. However, whether the successful existence of the SXT/R391 ICEs is related exclusively to the appearance of resistance determinants is inconclusive. Other genes found in the variable regions, including genes encoding unknown functions, may be related to the enhancement of the adaptability of ICEs^12^.

In this study, the fifteen ICEs were grouped into six clusters by the phylogenetic analysis and therefore may be representative of six different evolutionary origins. Six ICEs were placed in cluster I, these ICEs were identical to ICE*Pmi*Chn1, which is a recently reported ICE contained in *P. mirabilis* that was isolated from a faecal sample from chicken in Hubei, China, in 2013^17^. ICE*Pmi*Chn1 was a predominant SXT/R391 family member in clinical *Proteus* sources in China and probably experienced horizontal transmission from food to humans. Because the six ICEs and the ICE*Pmi*Chn1-containing strains were isolated during and after 2013 (Supplementary Tables S1 and S2), these ICEs may have appeared during the latest period of their evolution and then presented an extensive distribution in China. Three ICEs (cluster II in Fig. 1) with references including ICEs from *V. cholerae* strain ICDC-4210 (isolated from Jiangxi, China, in 1999)^31^ and *P. mirabilis* strain HI4320 (USA, 1986)^33^, indicated that the ICEs of this cluster may have appeared as early as 1986 and have been transmitted among different bacterial species and countries. Similarly, ICE*Pvu*CHN2213 in cluster III contained in a *P. vulgaris* strain isolated from food was closely related to the ICE MED64^25^ (from the Aegean Sea near Lebanon, 2000), which was between clusters I and II. Interestingly, five novel ICEs in this study were grouped into three distinctive clusters (IV, V and VI in Fig. 1) with no references in the three clusters, which might indicate that they were independently acquired by *Proteus* strains. Generally, the phylogenetic analysis suggested that ICEs could be transmitted among *Proteus*, *V. cholerae* and other *Enterobacteriaceae* and might share a common ancestor although they evolved independently. The acquired ICEs in *Proteus* were not species-specific under certain conditions; for example, to adapt to the environment and to facilitate survival under selective pressure, *Proteus* strains are able to acquire ICEs from different sources and evolutionary stages^35^.

The majority of known SXT/R391 ICEs contain four types of antibiotic genes (*strA*/*B*, *sul2*, *floR* and *dfrA*) that confer resistance to streptomycin, sulfamethoxazole, chloramphenicol and trimethoprim, respectively. R391 contains a kanamycin-encoding gene^11^ and other new antibiotic resistance genes of known SXT/R391 ICEs carried have also been found (e.g. the cephalosporin resistance gene *bla*_CMY-2_^16^ and rifampicin resistance gene)^13^. In this study, isolates positive for resistant related genes at SXT/R391 ICEs were not always consistent with their phenotypic resistance (Table 1), this result indicated the phenotypic resistance may not be associated with genes encoded by ICEs, determinants of other mobile genetic elements, like plasmid^36^, transposon^37^ and genomic island^38^ within *Enterobacteriaceae*, are also mediated drug resistance. However, the role of ICEs in the acquisiton and transmission of antibiotic resistance should not be neglected, our study have displayed high transfer ability of ICEs from *Proteus* isolates to recipient cells. In addition, the 15 SXT/R391 ICEs carried not only the four common resistance genes mentioned above, nine SXT/R391 ICEs also carried a class A β-lactamase gene. In contrast, no dominant β-lactamase genes are carried by ICE*Pmi*Chn1^17^, suggesting that the class A β-lactamase gene might have been obtained via horizontal gene transfer or recombination. In summary, our study reported multi-drug resistance, including the increasing prevalence of class A β-lactamase-producing *P. mirabilis*, which is consistent with the trends in Japan^16^, Taiwan^39^ and other reports^24^,^40^. In conclusion, our results present abundant genetic diversity and multidrug resistance of ICEs carried by *Proteus* strains from both food sources and diarrhoeal patients. The SXT/R391 ICEs could be transferred between *Proteus* and other *Enterobacteriaceae*, thereby conferring resistance to the host and facilitating bacterial survival in the environment. Therefore, we need to strengthen the continuous monitoring of antimicrobial resistance and related mobile elements among *Proteus* isolates.

## Additional Information

**How to cite this article**: Li, X. *et al.* SXT/R391 integrative and conjugative elements in *Proteus* species reveal abundant genetic diversity and multidrug resistance. *Sci. Rep.* **6**, 37372; doi: 10.1038/srep37372 (2016).

**Publisher’s note:** Springer Nature remains neutral with regard to jurisdictional claims in published maps and institutional affiliations.

## Supplementary Material

Supplementary Information

## Figures and Tables

**Table 1:** Genomic features of the ICEs, antibiotic resistance patterns and transfer frequency of ICE-contained strains in this study.

Strain ID	Location	Species	Sample source	Year of isolation	ICE name	ICE length (bp)	G + C (%)	No. of predicted CDSs (RAST)	Antibiotic resistance phenotype	Transfer frequency	SXT/R391 ICE gene				
											*dfhR*	*floR*	*strB*/*A*	*sul2*	*tetA*/*R*
08MAS2213	Maanshan	*P. vulgaris*	food	2008	ICE*Pvu*CHN2213	94,340	46.8	92	AMP-AZM-CHL-CFZ-SAM-STR-SXT-SUL	1.0 × 10^−5^	−	+	+	+	−
08MAS1586	Maanshan	*P. mirabilis*	food	2008	ICE*Pmi*CHN1586	99,355	46.6	85	AMP-AZM-CHL-CIP-SAM-STR-SXT-SUL-**TCY**	2.5 × 10^−2^	+	+	+	+	−
09MAS2407	Maanshan	*P. mirabilis*	stool	2009	ICE*Pmi*CHN2407	97,078	47	95	AMP-AZM-**CHL**-CFZ-CIP-KAN-**STR-SXT-SUL**-TCY	9.5 × 10^−4^	−	−	−	−	+
09MAS2410	Maanshan	*P. mirabilis*	stool	2009	ICE*Pmi*CHN2410	93,537	46.5	86	AMP-AZM-**CHL**-CFZ-CIP-KAN-**STR-SXT-SUL**-**TCY**	NT	−	−	−	−	−
09MAS2416	Maanshan	*P. mirabilis*	stool	2009	ICE*Pmi*CHN2416	92,556	46.7	86	AMP-AZM-**CHL**-CFZ-CIP-GEN-KAN-**STR-SXT-SUL**-**TCY**	NT	−	−	−	−	−
MD20140901	Beijing	*P. mirabilis*	stool	2014	ICE*Pmi*CHN901	89,493	47.3	88	AMP-AZM-ATM-AMK-CHL-CXM-CFZ-CRO-CIP-GEN-SAM -KAN-STR-SXT-SUL-**TCY**	NT	+	+	+	+	−
MD20140902	Beijing	*P. mirabilis*	stool	2014	ICE*Pmi*CHN902	89,096	47.1	86	AMP-AZM-ATM-AMK-CHL-CXM-CFZ-CRO-CIP-SAM-KAN-STR-SXT-SUL-**TCY**	NT	+	+	+	+	−
MD20140903	Beijing	*P. mirabilis*	stool	2014	ICE*Pmi*CHN903	89,644	47.3	86	AMP-AZM-AMK-CHL-CXM-CFZ-CRO-CIP-SAM -KAN-STR-SXT-SUL-**TCY**	NT	+	+	+	+	−
MD20140904	Beijing	*P. mirabilis*	stool	2014	ICE*Pmi*CHN904	94,942	47.7	99	AMP-AZM-ATM-AMK-CHL-CXM-CFZ-CRO-CIP-FEP-SAM -KAN-STR-**SXT**-SUL-TCY	NT	−	+	+	+	+
MD20140905	Beijing	*P. mirabilis*	stool	2014	ICE*Pmi*CHN905	94,956	47.8	98	AMP-AZM-ATM-AMK-CHL-CXM-CFZ-CRO-CIP-GEN-SAM -KAN-STR-**SXT**-SUL-TCY	NT	−	+	+	+	+
TJ1809	Tianjin	*P. mirabilis*	stool	2013	ICE*Pmi*CHN1809	76,218	47.1	67	AMP-AZM-**CHL**-CIP-KAN-**SXT-SUL**-**TCY**	4.0 × 10^−5^	−	−	−	−	−
TJ3237	Tianjin	*P. mirabilis*	stool	2013	ICE*Pmi*CHN3237	87,215	46.8	74	AMP-AZM-CFZ-**SXT-SUL**-**TCY**	NT	−	−	−	−	−
TJ3277	Tianjin	*P. mirabilis*	stool	2013	ICE*Pmi*CHN3277	104,175	46.7	53	AMP-AZM-CFZ-SXT-SUL-TCY	NT	NT	NT	NT	NT	NT
TJ3300	Tianjin	*P. mirabilis*	stool	2013	ICE*Pmi*CHN3300	108,335	46.7	98	AZM-CHL-STR-SXT-SUL	NT	+	+	+	+	−
TJ3335	Tianjin	*P. mirabilis*	stool	2013	ICE*Pmi*CHN3335	89,996	47.8	84	AZM-CHL-KAN-STR-**SXT**-SUL-**TCY**	5.0 × 10^−6^	−	+	+	+	−

Abbreviations: AMP: ampicillin, AZM: azithromycin, CFZ: cefazolin, CXM: cefuroxime, CRO: ceftriaxone, FEP: cefepime, CTT: cefotetan, ATM: aztreonam, IPM: imipenem, SAM: ampicillin-sulbactam, TZP: piperacillin-tazobactam, AMK: amikacin, GEN: gentamycin, KAN: kanamycin, STR: streptomycin, SUL: sulfamethoxazole, SXT: trimethoprim-sulfamethoxazole, CIP: ciprofloxacin, CHL: chloramphenicol, TCY: tetracycline. Abbreviations in bold indicated positive resistance phenotype but negative for their gene at ICE. “+”: positive, “−”: negative, “NT”: not test.

**Table 2:** BLASTn searches against GenBank of the 15 SXT/R391-like ICEs.

SXT	Description of ICEs from species	Max score	Total Score (bits)	Query cover	Identity	Accession
ICE*Pvu*CHN2213	*Alteromonas macleodii* str. MED64’E	1.30E + 05	1.71E + 05	86%	99%	CP004848.1
	*Vibrio cholerae* strain AHV1003	1.13E + 05	1.74E + 05	92%	99%	KT151663.1
	*Vibrio cholerae* strain TSY216	1.13E + 05	1.74E + 05	92%	99%	CP007653.1
ICE*Pmi*CHN1586	*Vibrio cholerae* strain ICDC-2605	57943	1.95E + 05	95%	100%	KT151661.1
	*Vibrio cholerae* strain ICDC-1605	57943	1.95E + 05	95%	100%	KT151656.1
	*Vibrio cholerae* strain ICDC-143	57943	1.95E + 05	95%	100%	KT151654.1
ICE*Pmi*CHN2407	*Proteus mirabilis* ICE*Pmi*Chn1	32073	1.08E + 05	64%	98%	KT962845.1
	*Vibrio cholerae* O37 strain MZ03	29263	73099	46%	96%	JQ345361.1
	*Vibrio alginolyticus* strain HN396	26856	84281	53%	96%	KT072770.1
ICE*Pmi*CHN2410	*Proteus mirabilis* ICE*Pmi*Chn1	32058	1.08E + 05	66%	98%	KT962845.1
	*Vibrio cholerae* O37 strain MZ03	29263	73099	47%	96%	JQ345361.1
	*Vibrio alginolyticus* strain HN396	26856	84281	55%	96%	KT072770.1
ICE*Pmi*CHN2416	*Proteus mirabilis*, ICE*Pmi*Chn1	37102	1.08E + 05	67%	99%	KT962845.1
	*Vibrio cholerae* O37 strain MZ03	29263	73099	48%	96%	JQ345361.1
	*Vibrio alginolyticus* strain HN396	26856	84281	55%	96%	KT072770.1
ICE*Pmi*CHN901	*Proteus mirabilis* ICEPmiChn1	42494	1.32E + 05	77%	99%	KT962845.1
	*Vibrio cholerae* strain ICDC-2605	34481	1.26E + 05	73%	99%	KT151661.1
	*Vibrio cholerae* strain ICDC-1605	34481	1.26E + 05	73%	99%	KT151656.1
ICE*Pmi*CHN902	*Proteus mirabilis* ICE*Pmi*Chn1	40945	1.31E + 05	76%	99%	KT962845.1
	*Vibrio cholerae* strain wujiang-2	36490	1.14E + 05	69%	96%	KT151664.1
	*Vibrio cholerae* O1 str. KW3	36490	1.19E + 05	72%	96%	CP006947.1
ICE*Pmi*CHN903	*Proteus mirabilis* ICE*Pmi*Chn1	39305	1.33E + 05	77%	99%	KT962845.1
	*Vibrio cholerae* strain wujiang-2	36490	1.14E + 05	69%	96%	KT151664.1
	*Vibrio cholerae* O1 str. KW3	36490	1.20E + 05	72%	96%	CP006947.1
ICE*Pmi*CHN904	*Proteus mirabilis* ICE*Pmi*Chn1	42512	1.35E + 05	74%	99%	KT962845.1
	*Vibrio cholerae* strain wujiang-2	36490	1.12E + 05	64%	96%	KT151664.1
	*Vibrio cholerae* O1 str. KW3	36490	1.27E + 05	68%	96%	CP006947.1
ICE*Pmi*CHN905	*Proteus mirabilis* ICE*Pmi*Chn1	42505	1.37E + 05	75%	99%	KT962845.1
	*Vibrio cholerae* strain wujiang-2	36490	1.16E + 05	65%	96%	KT151664.1
	*Vibrio cholerae* O1 str. KW3	36490	1.22E + 05	68%	96%	CP006947.1
ICE*Pmi*CHN1809	*Providencia stuartii* strain ATCC 33672	25080	1.02E + 05	79%	97%	CP008920.1
	*Vibrio alginolyticus* strain HN437	19398	78001	62%	98%	KT072771.1
	*Vibrio cholerae* Ind4 ICE*Vch*ind4	19287	81235	64%	97%	GQ463141.1
ICE*Pmi*CHN3237	*Proteus mirabilis* strain HI4320	1.36E + 05	1.45E + 05	90%	99%	AM942759.1
	*Vibrio cholerae* strain ICDC-4210	1.36E + 05	1.65E + 05	96%	99%	KT151662.1
	*Vibrio cholerae* strain ICDC-2605	1.36E + 05	1.58E + 05	96%	99%	KT151661.1
ICE*Pmi*CHN3277	*Vibrio cholerae* strain ICDC-4210	6584	1.38E + 05	71%	99%	KT151662.1
	*Vibrio cholerae* strain ICDC-2605	6584	1.37E + 05	71%	99%	KT151661.1
	*Proteus mirabilis* strain HI4320	6584	1.35E + 05	70%	99%	AM942759.1
ICE*Pmi*CHN3300	*Vibrio cholerae* MJ-1236	72417	1.80E + 05	87%	99%	CP001485.1
	*Alteromonas mediterranea* strain U10	49808	91294	49%	97%	CP013930.1
	*Alteromonas mediterranea* strain UM8	49808	88620	47%	97%	CP013928.1
ICE*Pmi*CHN3335	*Proteus mirabilis* ICE*Pmi*Chn1	1.24E + 05	1.68E + 05	99%	99%	KT962845.1
	*Vibrio alginolyticus* strain A056	30134	1.28E + 05	81%	96%	KR231688.1
	*Vibrio cholerae* O37 strain MZ03	9198	77187	52%	96%	JQ345361.1
