# Supplementary material for: SXT/R391 integrative and conjugative elements in Proteus species reveal abundant genetic diversity and multidrug resistance
Source: Sci Rep. 2016 Nov 28;6:37372. doi: 10.1038/srep37372 (PMC5124997; doi:10.1038/srep37372)
Supplement: Supplementary Information [file srep37372-s1.pdf]

Supplementary Information:

SXT/R391 integrative and conjugative elements in *Proteus* species reveal abundant genetic diversity and multidrug resistance.

Xinyue Li[1,2], Yu Du[1,2], Pengcheng Du[3], Hang Dai[1,2], Yujie Fang[1,2], Zhenpeng Li[1,2], Na Lv[4], Baoli Zhu[4], Biao Kan[1,2], Duochun Wang[1,2*]

Supplementary Table S1. Twenty-four ICEs representative for the different evolutionary origins and species.

Strain name	ICEs name	Isolation place	Isolation source	Isolation year	Accession number
Alteromonas macleodii str. Aegean Sea MED64#	-	Aegean Sea of Lebanon	water	2000	CP004848.1
Alteromonas macleodii str. 'Ionian Sea UM7	-	Ionian Sea	water	-	CP004853.1
Photobacterium damselae subsp. Piscicida PC554.2	ICE*Pda*Spa1	Galicia, Spain	fish	-	AJ870986.2
Proteus mirabilis PM14C28	ICE*Pmi*Jpn1	Heibei, China	Chicken	2014	KT894734.1
Proteus mirabilis PM13C04#	ICE*Pmi*Chn1#	Hubei, China	Chicken	2013	KT962845.1
Proteus mirabilis HI4320	ICE*Pmi*Usa1	USA	-	1986	AM942759.1
Providencia alcalifaciens Ban1	ICE *Pal* Ban1	Bangladesh	-	1999	GQ463139.1
Providencia rettgeri R391	R391	Pretoria, South A frica	-	1967	AY090559.1
Providencia stuartii ATCC 33672#	-	-	-	-	CP008920.1
Shewanella sp. W3-18-1	ICE*Spu*PO1	Pacific Ocean	marine sediments	2000	CP000503.1
Vibrio alginolyticus A056-1	ICE*Val*A056-1	China	gill	-	KR231688.1
Vibrio alginolyticus HN396	ICE*Val*HN396	China	seawater	2008	KT072770.1
Vibrio alginolyticus HN492	ICE*Val*HN492	China	seawater	2008	KT072769.1
Vibrio alginolyticus A056-2	ICE*Val*A056-2	China	gill	2003	KR231689.1
Vibrio cholerae MJ-1236#	ICE*Vch*Ban9#	Bangladesh	-	1994	CP001485.1
Vibrio cholerae ICDC-4210 #	ICE*Vch*CHN4210#	China	Patient	1999	KT151662.1
Vibrio cholerae 2010EL-1786	SXT element	Haitian	Patient	2010	CP003069.1
Vibrio cholerae Ban5	ICE*Vch*Ban5	Bangladesh	-	1998	GQ463140.1
Vibrio cholerae Ind4	ICE*Vch*Ind4	Kolkata, India	-	1997	GQ463141.1
Vibrio cholerae Mex1	ICE*Vch*Mex1	San Luis Potosi, Mexico	-	2001	GQ463143.1
Vibrio cholerae MO10	SXT	Chennai, India	-	2002	AY055428.1
Vibrio parahaemolyticus UCM-V493	-	Spain	sediment sample	2002	CP007004.1
Vibrio cholerae ICDC-2605#	ICE*Vch*CHN2605#	Guizhou, China	fecal sample	1998	KT151661
Vibrio fluvialis H-08942	ICE*Vfl*Ind1	India	-	2002	GQ463144

"#": indicated the reference ICEs and/or strains used in supplementary Figure S1.

Supplementary Table S2. Primer information for amplicating antibiotic resistance genes in ICEs.

Target gene	Primer	Neucleotide Sequence(5'-3')	Amplicon size (bp)	Related antibiotics
strA	strA-F	TTGATGTGGTGTCCCGCAATGC	383	Streptomycin
	strA-R	CCAATCGCAGATAGAAGGCAA		
strB	strB-F	CGAGGCATTGCTCATCATTT	443	Streptomycin
	strB-R	TTCTCATTGCGGACACCTTTT		
floR	floR-F	CGACATCCTCGCTTCACTG	625	Chloramphenicol
	floR-R	GACGAAGAAGGTGCCCATAC		
sul2	sul2-F	TTTTCGGCATCGTCAACATAA	549	Sulfisoxazole
	sul2-R	AGAGCGAGGTTTCGGGAGC		
tetA	tetA-F	TTCCTGACGGGCTGTTTCCTT	600	Tetracycline
	tetA-R	TGCAATCCATGCCCACCCGTTCCAC		
dfrA1	dfrA1-F	CGAAGAATGGAGTTATCGGG	372	Trimethoprim
	dfrA1-R	TGCTGGGGATTTCAGGAAAG		

The primers were designed based on ICEs sequence of *Vibrio cholerae* MO10 (AY055428.1).

Supplementary Table S3.The predicted ORFs of the fifteen ICEs in this study.

ORF	ICE*Pvu*CHN2213	ICE*Pmi*CHN1586	ICE*Pmi*CHN2407	ICE*Pmi*CHN2410	ICE*Pmi*CHN2416	ICE*Pmi*CHN901	ICE*Pmi*CHN902	ICE*Pmi*CHN903
1	HP	HP	HP	HP	HP	HP	HP	HP
2	MutL	MutL	HP	HP	integrase	HP	HP	integrase
3	integrase	integrase	HP	HP	s002	integrase	integrase	s002
4	s002	s002	integrase	integrase	s003	s002	s002	s003
5	s003	s003	s002	s002	HP	s003	s003	HP
6	HP	HP	s003	s003	HP	HP	HP	HP
7	HP	HP	HP	HP	RumB	HP	HP	HP
8	HP	RumB	HP	HP	tnp	RumB	HP	RumB
9	RumB	tnp	RumB	RumB	tnpA	tnp	RumB	tnp
10	tnp	tnpA	tnp	tnp	HP	tnpA	tnp	tnpA
11	tnpA	tnpB	tnpA	tnpA	HP	tnpB	tnpA	tnpB
12	tnpB	HP	HP	HP	HP	DhfR	tnpB	DhfR
13	DhfR	DhfR	HP	HP	β-lactamase	tnpB	DhfR	tnpB
14	tnpB	tnpB	HP	HP	HP	s013	tnpB	s013
15	s013	s013	β-lactamase	β-lactamase	HP	FloR	s013	FloR
16	FloR	FloR	HP	HP	polymerase III	s015	FloR	s015
17	s015	s015	HP	HP	HP	tnpB	s015	tnpB
18	tnpB	tnpB	polymerase III	polymerase III	s026	StrB	tnpB	StrB

19	StrB	StrB	HP	HP	HP	StrA	StrB	StrA
20	StrA	StrA	s026	s026	Type I RM	Sul2	StrA	Sul2
21	Sul2	Sul2	HP	HP	HP	HP	Sul2	HP
22	tnpA	tnpA	Type I RM	Type I RM	HP	β-lactamase	HP	β-lactamase
23	RumB	RumB	HP	HP	Type I RM	HP	β-lactamase	HP
24	HP	HP	HP	HP	protein kinase	RumA	HP	polymerase III
25	RumA	RumA	Type I RM	Type I RM	HP	polymerase III	polymerase III	HP
26	polymerase III	polymerase III	protein kinase	protein kinase	helicase	HP	HP	s026
27	s025	s025	HP	HP	TraI	s026	s026	Type I RM
28	s026	s026	helicase	helicase	transposase	Type I RM	Type I RM	Type I RM
29	Type I RM	helicase	TraI	TraI	TraD	Type I RM	Type I RM	bstXI
30	Type I RM	Type II RM	transposase	HP	234	bstXI	bstXI	Type I RM
31	bstXI	helicase	TraD	TraD	s043	Type I RM	Type I RM	ATPase
32	Type I RM	helicase	234	234	HP	HP	ATPase	tnp
33	HP	TraI	s043	S043	Fic	tnp	tnp	HP
34	McrC	TraD	HP	HP	TraL	HP	HP	McrC
35	Mrr	234	Fic	Fic	TraE	McrC	McrC	Mrr
36	HP	s043	TraL	TraL	TraK	Mrr	Mrr	HP
37	HP	HP	TraE	TraE	TraB	HP	HP	HP
38	TraI	TraL	TraK	TraK	TraV	HP	HP	TraI
39	TraD	TraE	TraB	TraB	TraA	TraI	TraI	TraD
40	234	TraK	TraV	TraV	acetyltransferase	TraD	TraD	234
41	s043	TraB	TraA	TraA	HP	234	234	s043
42	tnp	TraV	acetyltransferase	acetyltransferase	s054	s043	s043	HP
43	HP	TraA	HP	HP	TraC	HP	HP	TraL
44	HP	Ynd	s054	s054	345	TraL	TraL	TraE
45	tnp	Ync	TraC	TraC	TrhF	TraE	TraE	TraK
46	HP	s054	345	345	TraW	TraK	TraK	TraB
47	TraL	TraC	TrhF	TrhF	TraU	TraB	TraB	TraV
48	TraE	HP	TraW	TraW	TraN	TraV	TraV	TraA
49	TraK	TrhF	TraU	TraU	HP	TraA	TraA	Ynd
50	TraB	TraW	TraN	TraN	helicase	Ynd	Ynd	Ync
51	TraV	TraU	HP	HP	Dns	Ync	Ync	s054
52	TraA	TraN	helicase	helicase	ISPpu12 tnpA	s054	s054	TraC
53	Ynd	HP	Dns	Dns	lspA	TraC	TraC	345
54	Ync	GTPases	ISPpu12 tnpA	lspA	CzcD	345	345	TrhF
55	s054	HP	lspA	CzcD	MerR	TrhF	TrhF	TraW
56	TraC	Dns	CzcD	s063	s063	TraW	TraW	TraU
57	HP	s063	MerR	HP	HP	TraU	TraU	TraN
58	TrhF	HP	HP	SSB	SSB	TraN	TraN	HP

59	TraW	SSB	HP	Bet	Bet	HP	HP	helicase
60	TraU	s065	Resolvase	HP	HP	helicase	helicase	Dns
61	TraN	HP	HP	s066	s066	Dns	Dns	ISPpu12 tnpA
62	HP	s066	HP	s067	s067	ISPpu12 tnpA	HP	s063
63	HP	s067	TetA	HP	HP	s063	SSB	HP
64	s063	HP	TetR	s068	s068	HP	Bet	SSB
65	HP	s068	s063	s069	s069	SSB	HP	Bet
66	SSB	s069	HP	s070	s070	Bet	s066	HP
67	Bet	s070	SSB	s071	s071	HP	s067	s066
68	HP	s071	Bet	HP	HP	s066	HP	s067
69	s066	HP	HP	primase	primase	s067	s068	s068
70	s067	primase	s066	s073	s073	HP	s069	s069
71	HP	s073	s067	TraF	TraF	s068	s070	s070
72	s068	HP	HP	TraH	TraH	s069	s071	s071
73	s069	endonuclease	s068	TraG	TraG	s070	HP	HP
74	s070	prcA	s069	MerR	MerR	s071	primase	primase
75	s071	TraF	s070	MerT	MerT	HP	s073	s073
76	HP	TraH	s071	MerP	MerP	primase	TraF	TraF
77	primase	TraG	HP	MerC	MerC	s073	TraH	TraH
78	s073	EexS	primase	MerA	MerA	TraF	TraG	TraG
79	HP	SetC	s073	EexR	EexR	TraH	EexS	EexS
80	HP	SetD	TraF	SetC	SetC	TraG	SetC	SetC
81	HP	s082	TraH	SetD	SetD	EexS	SetD	SetD
82	TraF	HP	TraG	s082	s082	SetC	s082	s082
83	TraH	s085	MerR	HP	HP	SetD	HP	HP
84	TraG	s086	MerT	s085	s085	s082	s085	s085
85	EexS	SetR	MerP	s086	s086	HP	s086	s086
86	SetC	-	MerC	SetR	SetR	s085	SetR	SetR
87	SetD	-	MerA	-	-	s086	-	-
88	s082	-	EexR	-	-	SetR	-	-
89	HP	-	SetC	-	-	-	-	-
90	s085	-	SetD	-	-	-	-	-
91	s086	-	s082	-	-	-	-	-
92	SetR	-	HP	-	-	-	-	-
93	-	-	s085	-	-	-	-	-
94	-	-	s086	-	-	-	-	-
95	-	-	SetR	-	-	-	-	-

ORF	ICE*Pmi*CHN904	ICE*Pmi*CHN905	ICE*Pmi*CHN1809	ICE*Pmi*CHN3237	ICE*Pmi*CHN3277	ICE*Pmi*CHN3300	ICE*Pmi*CHN3335
1	HP	HP	HP	HP	SetR	HP	HP
2	HP	HP	HP	integrase	s086	HP	HP
3	integrase	integrase	integrase	s002	HP	MutL	integrase
4	s002	s002	s002	s003	s082	integrase	s002
5	s003	s003	s003	HP	SetD	s002	s003

6	HP	HP	HP	HP	SetC	s003	HP
7	HP	HP	HP	RumB	HP	HP	HP
8	HP	HP	RumB	tnp	TraH	HP	RumB
9	RumB	RumB	RumA	tnpA	HP	RumB	tnp
10	tnp	tnp	IS4,transposase	tnpB	endonuclease	tnp	tnpA
11	tnpB	tnpA	polymerase III	HP	HP	tnpA	tnpB
12	s013	tnpB	HP	β-lactamase	HP	tnpB	s013
13	FloR	s013	HP	HP	HP	s013	FloR
14	s015	FloR	HP	polymerase III	s071	DhfR	s015
15	tnpB	s015	HP	HP	s069	HP	TetA
16	StrB	tnpB	HP	s026	s068	transposase	TetR
17	StrA	StrB	helicase	helicase	HP	s013	helicase
18	Sul2	StrA	Mrr	TypeII RM	HP	FloR	StrB
19	tnpA	Sul2	TraI	TypeIII RM	HP	HP	StrA
20	tnp	tnpA	TraD	helicase	s065	s015	Sul2
21	RumB	HP	234	TraI	SSB	tnpB	tnpI
22	RumA	β-lactamase	s043	TraD	HP	StrB	tnpII
23	HP	HP	HP	234	HP	StrA	MutL
24	β-lactamase	HP	TraL	s043	HP	Sul2	RumB
25	HP	polymerase III	TraE	HP	Dns	tnpA	RumA
26	HP	HP	TraK	TraL	HP	RumB	polymerase III
27	polymerase III	s026	TraB	TraE	TraN	RumA	HP
28	HP	Type I RM	TraV	TraK	TraU	HP	s026
29	s026	Type I RM	TraA	TraB	TraW	polymerase III	Type III RM
30	Type I RM	bstXI	HP	TraV	TrhF	HP	Type III RM
31	Type I RM	Type I RM	s054	TraA	345	s026	HP
32	bstXI	ATPase	TraC	Ynd	TraC	inner membrane protein	HP
33	Type I RM	tnp	345	Ync	Ync	cytoplasmic protein	HP
34	ATPase	HP	TrhF	s054	TraA	HP	TraI
35	tnp	McrC	TraW	TraC	TraV	HP	TraD
36	HP	Mrr	TraU	345	HP	RM II System	234
37	McrC	HP	TraN	TrhF	TraK	s038	s043
38	Mrr	HP	putative integrase	TraW	TraE	s039	HP
39	HP	TraI	transposase	TraU	TraL	ATPase	Transcriptional regulator
40	HP	TraD	HP	TraN	HP	TraI	TraL
41	TraI	234	s063	HP	s043	TraD	TraE
42	TraD	s043	HP	GTPases	TraD	234	TraK
43	234	HP	SSB	HP	TraI	s043	TraB
44	s043	TraL	Bet	Dns	Type II RM	HP	TraV
45	HP	TraE	HP	s063	helicase	TraL	TraA
46	TraL	TraK	s066	HP	HP	TraE	Ynd

47	TraE	TraB	s067	HP	HP	TraK	Ync
48	TraK	TraV	HP	SSB	polymerase III	TraB	s054
49	TraB	TraA	s068	Bet	β-lactamase	TraV	TraC
50	TraV	Ynd	s069	HP	HP	TraA	345
51	TraA	Ync	s070	s066	HP	Ynd	TrhF
52	Ynd	s054	s071	s067	s002	Ync	TraW
53	Ync	TraC	HP	HP	s003	HP	TraU
54	s054	345	primase	s068	-	helicase	TraN
55	TraC	TrhF	s073	s069	-	HP	HP
56	345	TraW	HP	s070	-	HP	helicase
57	TrhF	TraU	TraF	s071	-	type 4 fimbriae expression regulatory protein	Dns
58	TraW	TraN	TraH	HP	-	s054	ISPpu12 tnpA
59	TraU	HP	TraG	primase	-	TraC	s063
60	TraN	helicase	EexR	s073	-	345	HP
61	HP	Dns	SetC	HP	-	TrhF	SSB
62	helicase	ISPpu12 tnpA	SetD	endonuclease	-	TraW	Bet
63	Dns	lspA	s082	prcA	-	TraU	HP
64	ISPpu12 tnpA	CzcD	HP	TraF	-	TraN	s066
65	lspA	MerR	s085	TraH	-	prcA	s067
66	CzcD	HP	s086	TraG	-	exonuclease	HP
67	MerR	HP	SetR	EexS	-	serine protease	s068
68	HP	Resolvase	-	SetC	-	FtsH	s069
69	HP	HP	-	SetD	-	HP	s070
70	Resolvase	HP	-	s082	-	s063	s071
71	HP	TetA	-	HP	-	HP	HP
72	TetA	TetR	-	s085	-	SSB	primase
73	TetR	s063	-	s086	-	Bet	s073
74	s063	HP	-	SetR	-	HP	TraF
75	HP	SSB	-	-	-	s066	TraH
76	SSB	Bet	-	-	-	s067	TraG
77	Bet	HP	-	-	-	HP	EexS
78	HP	s066	-	-	-	s068	SetC
79	s066	s067	-	-	-	s069	SetD
80	s067	HP	-	-	-	s070	s082
81	HP	s068	-	-	-	s071	HP
82	s068	s069	-	-	-	HP	s085
83	s069	s070	-	-	-	primase	s086
84	s070	s071	-	-	-	s073	SetR
85	s071	HP	-	-	-	HP	-
86	HP	primase	-	-	-	DhfR	-
87	primase	s073	-	-	-	intIPac	-
88	s073	TraF	-	-	-	TraF	-

89	TraF	TraH	-	-	-	TraH	-
90	TraH	TraG	-	-	-	TraG	-
91	TraG	EexS	-	-	-	EexR	-
92	EexS	SetC	-	-	-	SetC	-
93	SetC	SetD	-	-	-	SetD	-
94	SetD	s082	-	-	-	s082	-
95	s082	HP	-	-	-	HP	-
96	HP	s085	-	-	-	s085	-
97	s085	s086	-	-	-	s086	-
98	s086	SetR	-	-	-	SetR	-
99	SetR	-	-	-	-	-	-

HP: Hypothetical protein

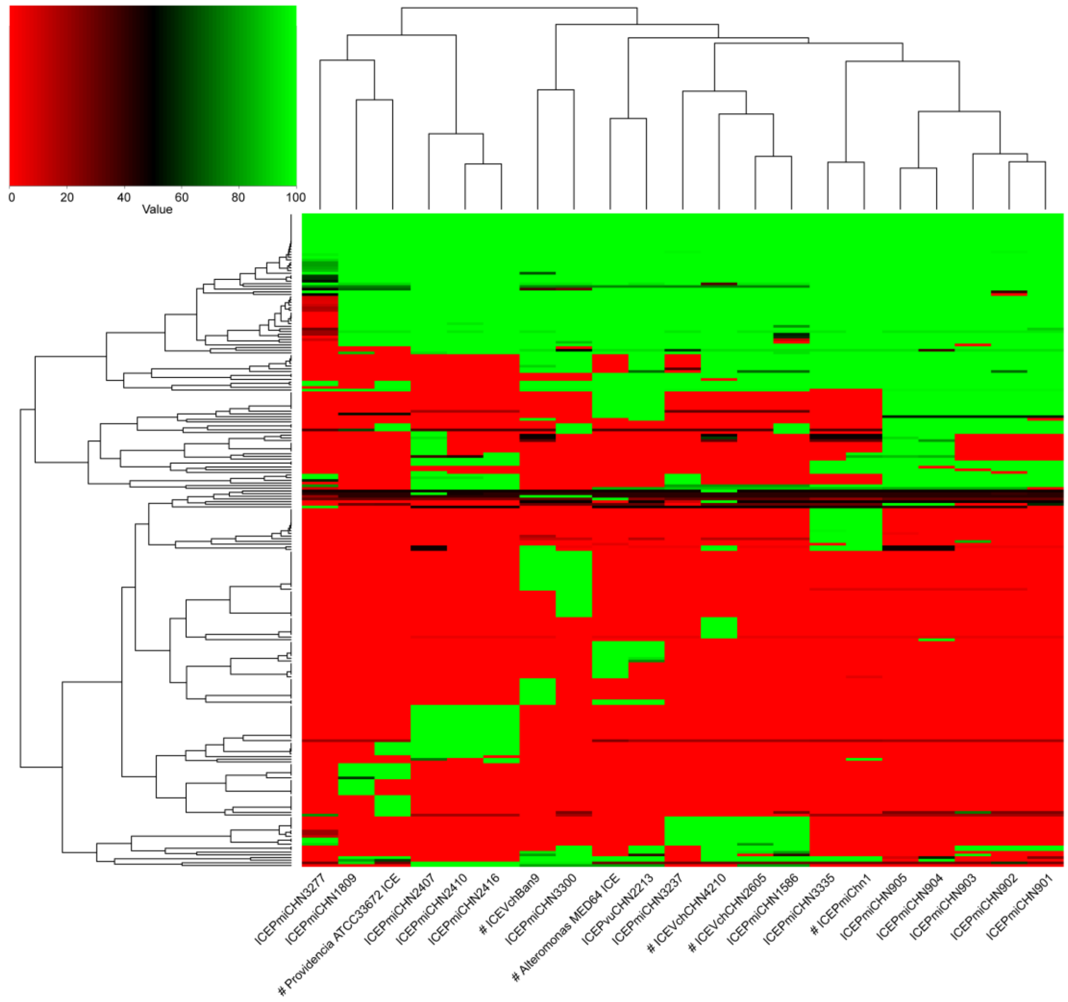

Supplementary Figure S1. Gene similarity heatmap of the fifteen ICEs in this study and six reference ICEs (indicated by “#”). The row cluster represents different ICEs and the column indicates annotated genes of each ICE (Table S1). The similarity value for each gene was depicted by colour with the legend indicated at the upper of the figure.
